# Impact of cognitive performance and negative symptoms on psychosocial functioning in Czech schizophrenia patients

**DOI:** 10.1038/s41537-023-00374-9

**Published:** 2023-07-17

**Authors:** L. Kalisova, J. Michalec, F. Dechterenko, P. Silhan, M. Hyza, M. Chlebovcova, M. Brenova, O. Bezdicek

**Affiliations:** 1 Department of Psychiatry, First Faculty of Medicine and General University Hospital in Prague, Charles University, Prague, Czech Republic; 2 Department of Psychology, Faculty of Arts, Charles University, Prague, Czech Republic; 3 Department of Psychiatry, Faculty Hospital, Ostrava, Czech Republic; 4 Department of Neurology and Centre of Clinical Neuroscience, First Faculty of Medicine and General University Hospital in Prague, Charles University, Prague, Czech Republic

**Keywords:** Schizophrenia, Human behaviour, Psychiatric disorders

## Abstract

Schizophrenia has a profound influence on the real-life functioning of patients. There are several factors inherent to the disease course affecting the level of psychosocial functioning. Our study focused on the impact of cognitive deficit and severity of negative symptoms (i.e., the experiential domain (avolition, asociality, and anhedonia) and the expressive domain (blunted affect and alogia)) to explore psychosocial functioning in schizophrenia. Schizophrenia patients (*n* = 211) were tested for the presence of cognitive impairment using the NIMH-MATRICS: Measurement and Treatment Research to Improve Cognition in Schizophrenia Consensus Cognitive Cattery (MCCB; MATRICS Consensus Cognitive Battery) and the extent of negative symptoms using the PANSS (PANSS; Positive and Negative Syndrome Scale—selected items). The level of psychosocial functioning was measured with the Personal and Social Performance Scale (PSP). The path analysis using three regression models was used to analyse variables influencing psychosocial functioning (PSP). One of these models analyzed influence of cognitive functioning (MCCB) and negative schizophrenia symptoms (PANSS selected items reflecting expressive and experiential deficits) as predictors and NART/CRT and disease length as confounders. R^2^ was 0.54. The direct effect of the MCCB (*β* = 0.09) on the PSP was suppressed by the strong effect of the negative symptoms (*β* = −0.64). The presence of cognitive deficits and negative symptoms in our sample of schizophrenia patients significantly influences the level of their psychosocial functioning, a key factor in remission and recovery.

## Introduction

Schizophrenia is a severe long-term psychiatric disorder characterized by diverse psychopathology and individual disease course. Schizophrenia can profoundly influence the real-life functioning of affected persons and can also harm their relatives and caregivers. Its treatment and consequences pose a burden to healthcare and social systems^1,2^. Patients with schizophrenia have difficulties obtaining and maintaining jobs and struggle to live independently and have relationships outside of the scope of family or caregivers. Only around 10–20% of patients work full- or part-time, which shows severe social functioning impairment^3^.

The principal aim of schizophrenia treatment is to achieve recovery. Recovery is a long-term multidimensional individually variable process, which is difficult to clearly define. According to a review of Vita et al., recovery can be assessed objectively and also from a subjective personal point of view. Objective and subjective domains of recovery may influence one another^4^. Objective domain comprises symptoms’ severity and a level of patients’ functioning. Subjective perspectives contributing to recovery include individual resilience, satisfaction with quality of life (with social inclusion and the social situation in general), personal confidence and hope, empowerment, the presence of self-stigmatization by mental illness, etc^4^.

Due to the variability of functioning over time, the interaction of both approaches mentioned above and the additional influence of an individual’s social environment, it is difficult to define and assess the level of recovery^5–7^.

Generally, it can be understood that a treatment goal could be the achievement of a state in which a person becomes involved in a subjectively satisfactory life in which they have a meaningful role allowing them to fit into society^8^.

Recovery rates seem to show an incremental trend, possibly due to better and more individualized pharmacological treatment and a variety of psychosocial approaches in recent decades. Patients have a higher probability of achieving recovery after the first episode of schizophrenia in comparison to patients with remittent episodes. The percentage of patients achieving recovery is still low, reaching 13–50% according to different studies^6,9^.

Full remission of symptoms is not essential for functional remission or recovery, but the reduction of psychopathology is a precondition to it. As defined by The Remission in Schizophrenia Working Group, remission requires a minimization of schizophrenia symptoms to the level that they do not interfere with behaviour in order to achieve adequate social functioning and symptomatology stabilisation, which should last at least six months^10^. Psychopharmacological treatment has been improving lately with several new antipsychotics, which seem to be promising not only in terms of the treatment of positive psychotic symptoms but also of negative, cognitive, and depressive symptoms^11,12^. Unfortunately, the pharmacological effect on psychotic symptoms other than positive is still insufficient^13,14^. The treatment of cognitive deficits and negative symptoms still has unmet needs^14,15^.

Negative symptoms and cognitive deficits that are present throughout life to a various extent in a high percentage of schizophrenia patients are usually key predictors of functional outcomes^16–26^.

Cognitive impairment has been considered nowadays as a core feature of the illness. Various works on this topic have confirmed that more than 80% of patients with schizophrenia suffer from various degrees of cognitive deficit, which markedly influence daily functioning^14,16–18^. Cognitive impairment can be detected before the appearance of first-episode symptoms (at prodromal state) and to some extent also in the first degree of healthy relatives^27,28^. A deficit of cognitive performance can be found in several neurocognitive domains and also in social cognition in schizophrenia patients^14^. The NIMH-MATRICS (The National Institute of Mental Health—Measurement and Treatment Research to Improve Cognition in Schizophrenia) initiative formed a consensus resulting from a broad-based multidisciplinary process and review of factor studies and identified six neurocognitive domains that are most affected in schizophrenia patients—verbal and visual memory, attention, speed of processing, working memory, and executive functions^29,30^. Social cognition includes the identification and interpretation of emotions (based on facial expressions, gestures, and body movement signals) and the further analysis of emotional motives and thoughts, mentalizing (understanding the intentions, feelings and purposes of other people), and understanding social situations and interactions, and also covers emotional regulation and meta-cognition in a social context. A panel of international experts reviewed the results from the NIMH-SCOPE (Social Cognition Psychometric Evaluation) study and agreed on four main domains of social cognition that are impaired in schizophrenia—emotional processing (reflecting the worsening of the ability to perceive and use emotional information), social perception (the inability to understand social cues), theory of mind (mainly difficulties in mentalizing) and attribution styles/bias (corresponding to attributing hostile intentions in ambiguous social situations)^14,31,32^. Social cognition is partially independent of other neurocognitive constructs and can have an even stronger impact on everyday functioning than neurocognition^32–36^.

Negative symptoms are present in around 50–60% of schizophrenia patients even before the first episode of the disease and tend to be highly persistent during the clinical stability period as well^37^. The occurrence of negative symptoms has an important impact on achieving remission and quality of life and functioning^38^.

Negative symptoms are heterogenous, but the EPA guidelines on the negative symptoms of schizophrenia recently defined five main domains of negative symptoms based on the NIMH-MATRICS consensus statement: anhedonia, avolition, blunted affect, alogia, and asociality^39,40^. Negative symptoms can be grouped into two interconnected but independent domains/deficits—emotional expression and emotional experience^41^. The expression of emotions is presented as blunted affect and alogia and mainly affects active relationships. Emotional experience, including avolition, anhedonia and asociality, mirrors a lack of motivation to attend activities and social interaction and is linked to poorer functional outcomes^19–25,42^.

There is also a need to distinguish primary negative symptoms (part of the disease course) from secondary symptoms (induced by the adverse effects of pharmacological treatment, influenced by positive symptoms, depression, social deprivation and comorbid substance abuse)^39^. The degree of negative symptoms interferes with social functioning and predicts future psychosocial functioning even better than positive symptoms^38,40,43,44^. Our study aims to find out the extent to which cognitive functioning and the persistence of negative symptoms (emotional experience and expression) can influence psychosocial functioning when taking into account the length of illness, age and estimated premorbid intellectual capacity as confounders.

## Methods

### Participants

The sample comprised patients between 18 and 65 years of age, with a diagnosis of schizophrenia according to ICD-10 (F20.x) regardless of the length of illness. An ICD-10 diagnosis of schizophrenia was made by a clinical psychiatrist specialising in using a clinical interview based on M.I.N.I. (The Mini-International Neuropsychiatric Interview) for psychotic disorders studies.

All patients were stable in outpatient psychiatric care. Stabilisation was confirmed by an out-patient psychiatrist and also assessed by a recruiting psychiatrist before inclusion in the study. At the time of the assessment, the included patients were without acute psychotic symptoms (rated less than 3 on the PANSS-P), without psychoactive substance intoxication, and were stabilised on antipsychotic medication (i.e., olanzapine equivalents)^45^. Participants were recruited from patients previously hospitalised at the Departments of Psychiatry, at the General University Hospital in Prague and University Hospital in Ostrava.

Participants were excluded from the study if they had a history of CNS trauma, a neurological disorder or premorbid intellectual disability. Patients with substance dependence (except for nicotine) were also excluded.

All participants signed written informed consent. The ethical committee of the General University Hospital in Prague and the University Hospital in Ostrava approved the study.

### Assessments

#### Psychiatric evaluation

Basic sociodemographic (age, gender, employment, length of education) and clinical data (length of illness, current medication) were gathered. Sample characteristics are displayed in Table 1.

**Table 1:** Demographic and clinical characteristics of the sample (*n* = 211).

Variable	Values
Age (years)	34.18 (9.98)
Education (years)	13.40 (2.54)
Race (Caucasian, %)	211 (100%)
Sex (male, %)	166 (80%)
Handedness (right, %)	174 (93%)
Employment (employed/unemployed/disability, %)	40/61/66 (24%/37%/40%)
MCCB (Composite *T*-score)	35.07 (10.04)
MCCB domain: Speed of Processing	32.12 (13.50)
MCCB domain: Attention/Vigilance	34.08 (10.66)
MCCB domain: Working Memory	36.25 (12.12)
MCCB domain: Verbal Learning	36.96 (7.65)
MCCB domain: Visual Learning	40.02 (13.52)
MCCB domain: Reasoning and Problem Solving	40.42 (9.81)
MCCB domain: Social Cognition	32.95 (11.62)
PANSS total score	67.93 (14.11)
PANSS expression	11.42 (3.47)
PANSS experience	9.24 (2.91)
PANSS depression	5.66 (2.01)
Olanzapine (equivalency ratio)	21.67 (8.56)
PSP	51.84 (11.52)
PSP-A	2.83 (0.79)
PSP-B	2.47 (0.82)
PSP-C	1.18 (1.07)
PSP-D	0.31 (0.58)
NART/CRT (mean errors/premorbid IQ)	26.31 (11.58)/111 IQ (100–122 IQ)
Length of disease (months)	91.76 (101.64)

*MCCB* MATRICS Consensus Cognitive Battery, *NART/CRT* National Adult Reading Test/Czech Reading Test, *PANSS* Positive and Negative Syndrome Scale (PANSS expression items: Blunted Affect (N1), Poor Rapport (N3), Lack of Spontaneity (N6), and Motor Retardation (G7); PANSS experience items: Emotional Withdrawal (N2), Passive Social Withdrawal (N4) and Active social avoidance (G16), *PSP* Personal and Social Performance scale.

The presence and severity of symptoms of schizophrenia were assessed using the Positive and Negative Syndrome Scale (PANSS). The PANSS includes 30 items assessing the severity of psychopathology, scaled from 1 (an absent symptom) to 7 (a symptom of extreme severity). For the assessment of negative symptoms, the two-factor model that differentiates emotional experience from emotional expression was used^46,47^, but according to the current conceptualisation of negative symptoms^39^. The PANSS expression domain included the following items: PANSS Blunted Affect (N1), Poor Rapport (N3), Lack of Spontaneity (N6); PANSS experience domain items included: Emotional Withdrawal (N2) and Passive Social Withdrawal (N4).

To exclude the effect of possible depression as a secondary negative symptom, we also included depression items (measured solely by the PANSS item Depression (G6), or by a depression subscale—a combination of items: Somatic Concerns (G1), Anxiety (G2), Guilt Feelings (G3) and Depression (G6))^48^ in our analysis.

The dose equivalence estimate of antipsychotics was based on olanzapine equivalents^49^.

The level of psychosocial functioning was measured with the Personal and Social Performance Scale (PSP). The PSP assesses four domains of psychosocial functioning (A, socially useful activities; B, personal; C, social relationships and self-care; and D, disturbing and aggressive behaviour) on a scale from 1 to 100, where higher scores indicate better psychosocial functioning^50^.

Psychiatric scales (PANSS and PSP) were assessed by experienced psychiatrists specialising in general psychiatry officially trained in PANSS and PSP (within previous research projects). PANSS and PSP assessments also included repeated interrater reliability testing at the beginning of the study and when the interreliability coefficient was 0.8.

#### Neuropsychological assessment

The Czech version of the MATRICS (NIMH-MATRICS: Measurement and Treatment Research to Improve Cognition in Schizophrenia) consensus cognitive battery MCCB^51^ was administered during one assessment to those who met the inclusion and exclusion criteria. The Czech academic research translation of the MCCB is a full-fledged adaptation of the original US version of the battery including culturally adapted normative data^52–55^. A translation and back-translation and feasibility studies of the Czech MCCB version were done previously^53,56^. The MCCB consists of 10 test measures that cover seven cognitive domains: Speed of processing (Brief Assessment of Cognition in Schizophrenia, Symbol Coding (BACS-SC), Category Fluency (animals), and Trail Making Test, Part A (TMT-A)); Attention/Vigilance (Continuous Performance Test, Identical Pairs (CPT-IP)); Working memory verbal domain (Letter-Number Span; LNS) and non-verbal domain (Wechsler Memory Scale, Third Revision Spatial Span (WMS-III-SS)); Verbal learning (Hopkins Verbal Learning Test-Revised (HVLT-R)); Visual learning (Brief Visuospatial Memory Test-Revised (BVMT-R)); Reasoning and problem solving (Neuropsychological Assessment Battery, Mazes (NAB-Mazes)); and Social cognition (Mayer-Salovey-Caruso Emotional Intelligence Test, Managing Emotions (MSCEIT-ME)), which have been standardised with normative data developed for the US population^29,30^.

NART/CRT (National Adult Reading Test Czech Version/Czech Reading Test) is a performance-based reading measure of premorbid intellectual functioning that consists of 50 irregular words^57,58^.

Cognitive assessment was carried out by experienced psychologists extensively trained in the method of MCCB, who also participated in the normative MCCB study in the Czech Republic.

### Statistical analysis

Data were analysed in statistical software R (R Core Team, 2019). First, we report the correlation matrix for all measured variables. We adjusted the p-values for multiple comparisons using Holm correction. Our main analysis proceeded in two steps: first, we performed a regression analysis between negative symptoms, cognition, and functioning; second, the effects of schizophrenia on cognition across the age range were scrutinised.

We modelled the path analysis using three regression models to capture the influence of cognitive functioning (MCCB) and two negative schizophrenia domains (PANSS expression and experience) on psychosocial functioning including all four subscales (PSP/PSP-A through D) with confounders of premorbid intelligence (NART/CRT) and length of the disease. First, we ran three regression models: one was predicting the MCCB, while the remaining two were predicting PANSS expression and PANSS experience domain scores. All three models were using NART/CRT and length of disease as predictors. Then we predicted the PSP (and four subscales) using NART/CRT, length of disease, the PANSS (expression/experience) and the MCCB. All variables were z-transformed before they were used in the regressions, resulting in standardised *β* coefficients. Because we ran 10 models (5 different outcome variables—PSP and four subscales, 2 different PANSS predictors), we corrected the significance of the estimated *β* coefficients using Holm correction. To quantify model fit, we report the adjusted R^2^, which penalises the models with more predictors. Alternatively, we also computed additional measures (AIC – Akaike information criterion; BIC - Bayesian information criterion), but the implications for model comparison were similar and thus we report the adjusted R^2^ only.

## Results

The correlation between variables is visualised in Table 2. Both PANSS negative domain (experiential and expressive) subscales were highly correlated (*r* = 0.87), which resulted in similar estimates in all following models. In general, both PANSS negative domain subscales were negatively correlated with PSP (*r* = −0.67, −0.71) and positively correlated with PSP-A, B and C subscales (*r* ≥ 0.50). Moreover, the correlation of negative symptoms with PSP was similar when we controlled the possible effect of confounding variables (depression—PANSS depression subscale items G6, G1, G2, G3; positive symptoms—PANSS items P1 - delusions, P2 - conceptual disorganisation, P3 - hallucinatory behaviour) for both PANSS experience (*r*s = −0.71 to −0.65) and PANSS expression (*r*s = −0.67 to −0.62). This pattern was also observed for correlation with the length of disease (but to a smaller extent). For the MCCB, the pattern was similar for the PSP and subscales A, B and C, but in this case, PSP-D was also significant.

**Table 2:** Correlation between variables.

	MCCB	PANSS expression	PANSS experience	PSP	PSP-A	PSP-B	PSP-C	PSP-D	NART
PANSS expressions	−0.36^***^	-							
PANSS experience	−0.35^***^	0.88^***^	-						
PSP	0.34^***^	−0.71^***^	0.67^***^	-					
PSP-A	−0.31^***^	0.61^***^	0.56^***^	−0.83^***^	-				
PSP-B	−0.28^**^	0.58^***^	0.55^***^	−0.77^***^	0.58^***^	-			
PSP-C	−0.19	0.55^***^	0.50^***^	−0.62^***^	0.48^***^	0.45^***^	-		
PSP-D	−0.21^*^	0.12	0.08	−0.35^***^	0.26^**^	0.30^***^	0.29^***^	-	
NART/CRT	0.45^***^	−0.13	−0.20	0.24^*^	−0.19	−0.12	−0.17	−0.19	-
Length of disease	−0.10	0.39^***^	0.39^***^	−0.37^***^	0.31^***^	0.34^***^	0.37^***^	0.04	−0.05

*MCCB* MATRICS Consensus Cognitive Battery, *NART/CRT* National Adult Reading Test/Czech Reading Test, *PANSS* Positive and Negative Syndrome Scale, PANSS expression items: Blunted Affect (N1), Poor Rapport (N3), Lack of Spontaneity (N6), and Motor Retardation (G7); PANSS experience items: Emotional Withdrawal (N2), Passive Social Withdrawal (N4) and Active social avoidance (G16); *PSP*, Personal and Social Performance scale.

*P*-values were corrected using Holm correction.

^*^p < .05, ^**^p < .01, ^***^p < .001.

When predicting the MCCB composite score from NART/CRT and length of disease, we found that NART/CRT significantly predicted the MCCB score (*β* = 0.45, *p* < 0.001) while the length of disease did not (*β* = −0.07, *p* = 0.331) with the average model fit (adjusted R^2^ = 0.20). For the two models predicting the PANSS experience (or expression respectively) scores from NART/CRT and length of disease, the results were similar. We found that both variables significantly predicted the PANSS expression (NART/CRT: *β* = −0.17, *p* = 0.017, length of disease: *β* = 0.37, *p* < 0.001, adjusted R^2^ = 0.17) and experience (NART/CRT: *β* = −0.20, *p* = .003, length of disease: *β* = 0.37, *p* < 0.001, adjusted R^2^ = 0.18).

**Fig. 1:**
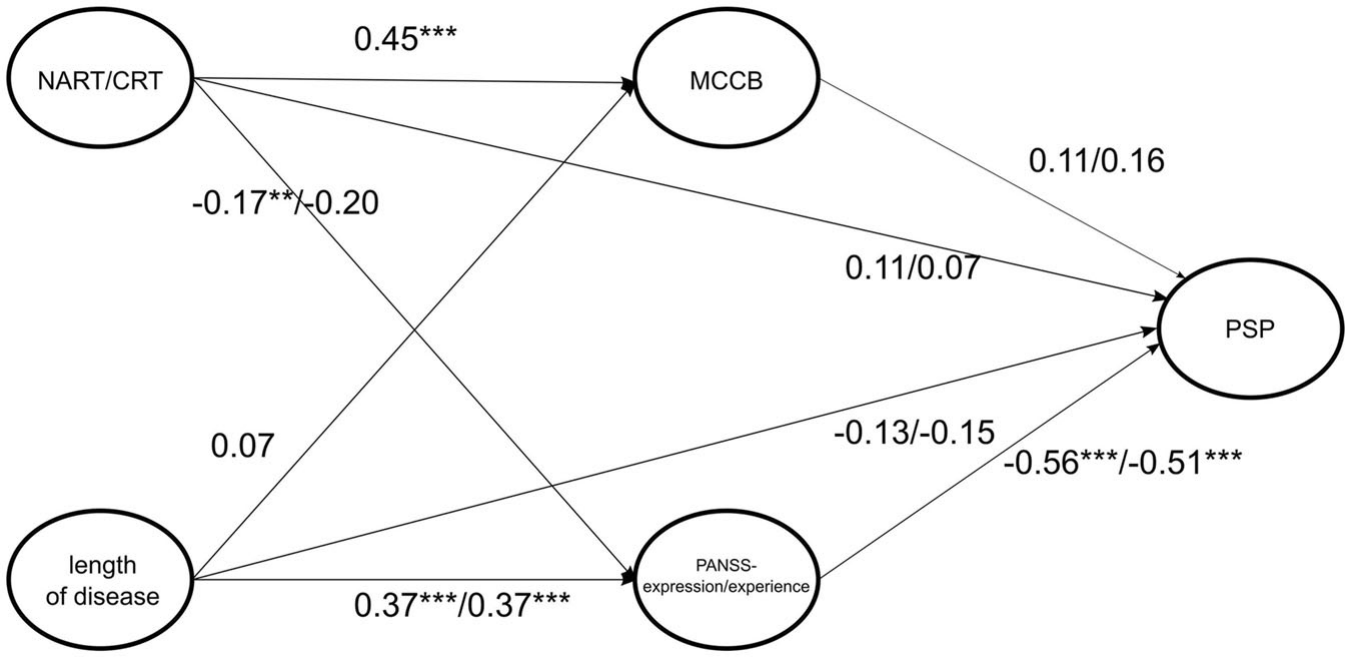
The path analysis using standardized regression coefficients depicting the influence of cognitive functioning (MCCB) and negative schizophrenia symptoms (PANSS expression/emotional) on psychosocial functioning (PSP) with the confounders of premorbid intelligence (NART/CRT) and length of the disease. MCCB MATRICS Consensus Cognitive Battery, PANSS Positive and Negative Syndrome Scale, PSP Personal and Social Performance Scale, NART/CRT National Adult Reading Test/Czech Reading Test.

The core results of predicting the PSP and its subscales from all remaining variables are visualized in Table 3 (similar results were obtained when both scales were used together forming PANSS negative scale, see Appendix, Table 5). In general, models behaved similarly irrespective of the PANSS negative domain subscales used. The best fit was observed for composite PSP in both PANSS expression (adjusted R^2^ = 0.48) and PANSS experience (adjusted R^2^ = 0.46), while it was lower for subscales A-C and both PANSS subscales (adjusted R^2^ = 0.28–0.35). For PSP-D, the model fit was poor for both PANSS expression and experience (adjusted R^2^ = 0.05 in both cases). We found that NART is not significant in any of the ten models (after the correction for multiple comparisons). Length of disease predicts PSP-C only (in both expression and experience subscales), while it is non-significant in the remaining subscales/full scale. The MCCB was non-significant in all five cases. On the other hand, both PANSS negative domain subscales were the strongest predictor, and it was significant in all subscales except for the PSP-D. The whole scheme depicting standardised regression coefficients is visualised in Fig. 1.

**Table 3:** Standardized coefficients for three models predicting PSP from the NART, length of disease, MCCB, and PANSS negative domains (emotional experience and expression).

PANSS predictor	Term	PSP	PSP-A	PSP-B	PSP-C	PSP-D
Expression	Intercept	−0.01	<0.01	0.05	0.08	0.03
	NART/CRT	0.11	−0.07	<0.01	−0.17	−0.14
	Disease length (months)	−0.13	0.12	0.15	0.23^*^	0.06
	MCCB (composite score)	0.11	−0.16	−0.14	0.07	−0.20
	PANSS- expression	−0.56^***^	0.45^***^	0.41^***^	0.43^***^	−0.05
	adj. R^2^	0.48	0.35	0.29	0.32	0.05
Experience	Intercept	−0.01	<0.01	0.05	0.09	0.03
	NART/CRT	0.07	−0.04	0.03	−0.15	−0.15
	Disease length (months)	−0.15	0.14	0.17	0.26^**^	0.06
	MCCB (composite score)	0.16	−0.20.	−0.18	0.01	−0.20
	PANSS experience	−0.51^***^	0.39^***^	0.38^***^	0.34^***^	−0.07
	adj. R^2^	0.46	0.32	0.28	0.28	0.05

*MCCB* MATRICS Consensus Cognitive Battery, *NART/CRT* National Adult Reading Test/Czech Reading Test, *PANSS* Positive and Negative Syndrome Scale PANSS expression items: Blunted Affect (N1), Poor Rapport (N3), and Lack of Spontaneity (N6); PANSS experience items: Emotional Withdrawal (N2), and Passive Social Withdrawal (N4), *PSP* Personal and Social Performance scale. ^*^p < .05, ^**^p < .01, ^***^p < .001; significance adjusted by Holm correction.

Each column corresponds to a model predicting different PSP subscales (or a composite score).

### Post-hoc analyses

To further explore the link between the variables, we improved the original model by modifying three relationships. First, to observe the possible exploratory effect of the MCCB on the PSP (including subscales), we removed the PANSS (expression or experience) predictor, leaving the models with NART, length of disease and MCCB variables. Second, when predicting the PANSS expression/experience using NART and length of disease, we also added the MCCB as a possible predictor. The third model added depression symptoms as an additional predictor of PSP scales.

When the PANSS subscale was removed from the predictors, the PSP was predicted by both length of disease (for all models with the exception of PSP-D) and MCCB (for PSP, PSP-A, and PSP-B; Table 4). Note that the adjusted R^2^ was smaller than in the case with the PANSS expression/experience subscales (the PSP with the PANSS expression/experience: adj. R^2^ = 0.48/0.46; the PSP without the PANSS negative scales: adj. R^2^ = 0.27).

**Table 4:** Standardized coefficients for three models predicting PSP from the NART, length of disease, and MCCB.

Term	PSP	PSP-A	PSP-B	PSP-C	PSP-D
Intercept	−0.04	0.03	0.07	0.11	0.02
NART/CRT	0.09	−0.06	0.02	−0.16	−0.14
Disease length (months)	−0.32^***^	0.27^***^	0.29^***^	0.38^***^	0.04
MCCB (composite score)	0.34^***^	−0.34^***^	−0.31^***^	−0.11	−0.17
adj. R^2^	0.27	0.21	0.18	0.2	0.05

*MCCB* MATRICS Consensus Cognitive Battery, *NART/CRT* National Adult Reading Test/Czech Reading Test. ^***^p < .001; significance adjusted by Holm correction.

Each column corresponds to a model predicting different PSP subscales (or a composite score).

For the second alternative model (predicting the PANSS expression/experience with the MCCB as an additional predictor), the length of disease and MCCB were significant predictors for both PANSS expression (length of disease: *β* = 0.34, *p* < 0.001; MCCB: *β* = −0.41, *p* < 0.001) and PANSS experience (length of disease: *β* = 0.34, *p* < 0.001; MCCB: *β* = −0.35, *p* < 0.001) while NART was non-significant in both cases (expression: *β* = 0.03, *p* = 0.703; experience: *β* = −0.04, *p* = 0.627). Note that in comparison to the original model, the adjusted R^2^ was larger (PANSS expression without the MCCB: adj. R^2^ = 0.17; with the MCCB: adj. R^2^ = 0.29; PANSS experience without the MCCB: adj. R^2^ = 0.18; with the MCCB: adj. R^2^ = 0.26) and NART/CRT was significant only in the model without the MCCB.

Finally, with the addition of depression symptoms, we obtained similar results–PSP was predicted by both negative symptoms (PANSS expression: *β* = −0.56, *p* < 0.001; PANSS experience: *β* = −0.51, *p* < 0.001), was predicted by length of disease (PANSS expression: *β* = −0.13, *p* = 0.040; PANSS experience: *β* = −0.15, *p* = 0.020), and was not predicted by NART (PANSS expression: *β* = 0.10, *p* = 0.126; PANSS experience: *β* = 0.07, *p* = 0.333). Depression symptoms were not significant in both models (PANSS expression: *β* = 0.07, *p* = 0.255; PANSS experience: *β* = 0.05, *p* = 0.390). The only difference was MCCB, which was not significant in the model with PANSS experience (*β* = 0.11, *p* = 0.140), but significant in the model with PANSS expression (*β* = 0.16, *p* = 0.032). However, after correcting for multiple testing, only the negative symptoms remain significant (*p*s < 0.001).

As visualised in Fig. 1, we show the model of the path analysis using three regression models with the influence of cognitive functioning (MCCB) and negative schizophrenia symptoms (PANSS expression/emotional) on psychosocial functioning (PSP) with the confounders of premorbid intelligence (NART/CRT) and length of the disease. We report estimates for both PANSS subscales by each arrow divided by a slash symbol.

Taken together, we were able to predict the PSP with other variables to a large extent (adj. R^2^ = 0.48). The PSP can still be predicted without the PANSS expression/experience, although the fit was worse.

## Discussion

The main goal of our study was to learn about the influence of cognitive impairment and presence of negative symptoms (emotional experience and expression) on psychosocial functioning. Over two hundred schizophrenia patients in a non-acute state were assessed. Length of illness, age and estimated premorbid intellectual capacity were analysed as confounders. The influence of depression and positive symptoms (as secondary negative symptoms) was tested and excluded.

As a result of our study, the model, where negative symptoms (both experience and expression deficit) and also cognitive deficit predict significantly psychosocial functioning is presented.

In our study sample, the impairment of cognitive performance was present in all cognitive domains of the MCCB. In all seven assessed domains cognitive deficit was found to be from 1–2 standard deviations below the mean; the MCCB composite score corresponded to 1.5 SD below the population mean. This finding corresponds with the typically stated cognitive deficit in patients with schizophrenia, which ranges from around a 0.75–1.5 standard deviation from healthy samples^31^. Our results do not show an association between cognitive performance and disease duration. Thus, our data are in line with previous studies in which the cognitive deficit is present during and after the first episode and then is stable throughout the disease course^22,28,31,33^.

Indeed, the association between premorbid intelligence level and cognitive performance, found in the current study, is not surprising. Premorbid intelligence has an indirect impact on psychosocial functioning through cognitive impairment due to schizophrenia. It could be presumed that patients with lower premorbid intellectual capacity, which is again impaired by the outbreak of schizophrenia, would have more difficulty in independent everyday functioning^59^.

This important link between cognitive performance and psychosocial functioning was also found in other studies; the effect of cognitive performance on functional outcome has a medium effect size for different domains and is even higher for the composite score^21,26,35,60^. In our study, cognitive deficit also influenced psychosocial functioning with medium predictive value; however, after taking into account negative symptoms (experience and expression deficits), the predictive influence of cognitive impairment was substantially lower. Some studies demonstrated in contradiction with our results a higher impact of cognitive impairment on function outcome than psychotic symptoms (negative and positive)^23,25^.

In our sample, the worst findings in cognitive performance were in social cognition, speed of processing and attention/vigilance domains. The deficit in social cognition in our sample is in line with previous research, in which social cognition is an important schizophrenia-specific factor, with an even more serious impact on psychosocial functioning than neurocognitive deficit^14,32–36^. A good level of social cognition allows for creating and maintaining interpersonal relationships and helps with social attribution and responsiveness, and also presumably helps to feel secure in social situations.

Social cognition influences significantly interpersonal relationships and the work environment^25^. According to the meta-analysis of Schmidt^36^, social cognition significantly mediates the indirect relationship between neurocognition and functional outcomes.

In our study, a two-factor model of negative symptoms was used. The PANSS scale has been used world-wide, but its official negative subscale also includes aspects that do not correspond to negative domains. According to EPA guidance on negative symptoms, we used the recommended concept of selected negative items from PANSS.

We decided to use a two-factor model to distinguish experiential deficits (avolition, asociality and anhedonia) and expressive (blunted affect, alogia) deficits. These domains could have different behavioural and neurobiological correlates and could have different future treatment approaches^38^.

In our patient sample, both domains of negative symptoms (emotional experience and expression) fundamentally predicted all domains of psychosocial functioning except PSP-D (disturbing or aggressive behaviour).

Based on our results, negative symptoms in both domains worsen with the duration of the illness. Length of disease had an indirect effect on psychosocial functioning in all PSP domains through negative symptoms (both experience and expression domains), except the domain of psychosocial functioning -domain social relationships and self-care (PSP-C), where the effect was direct.

According to EPA guidance on negative symptoms, we have to differentiate the influence of secondary negative symptoms^38^. In our sample there was not a significant influence of depression and acute positive symptoms (depression, delusion, conceptual disorganisation and hallucinations).

An international study from 11 European sites in almost 300 schizophrenia patients used the PANSS negative scale in correlation with different psychosocial functioning scales. This correlation was proven significant between the PANSS-N scale (full original) and different psychosocial functioning scales (correlation coefficients ranging from 0.63 to 0.80)^61^. Indeed, as found in our study, even low levels of negative symptoms were associated with the disruption of social functioning, whereas only severe cognitive impairment had a significant effect^62^.

The exact scope of the impact of negative symptoms and cognitive performance remains unanswered since there have been studies with inconsistent or contradictory results. However, all studies following negative and cognitive symptoms have confirmed that these factors are interconnected with everyday functioning. Some results indicate that negative and cognitive symptoms play independent and comparatively important roles^63^, while others assume dual pathway mechanisms in which motivation plays a decisive, direct role and cognition has an indirect role^64^. The interrelatedness between negative and cognitive symptoms is complicated by the fact that patients, based on failures due to reduced (cognitive) abilities, may adopt defeatist cognitive beliefs and a dysfunctional attitude and, therefore, be less motivated to attend everyday activities^64^. Our results regarding the aforementioned models are supportive of the third, dual pathway model.

Cognitive and negative symptoms can also affect different domains of everyday and psychosocial functioning in various ways. Okada et al. found that residential (e.g., self-care, the preparing of meals, and shopping) and vocational outcomes showed dual pathways while social (e.g., withdrawal, social relationships, and social participation) and recreational outcomes showed a single pathway^65^. Cognitive outcomes influenced residential activities like self-care or shopping; however, living and avolition mainly influenced recreational activities.

Our study shows that it is insufficient to account for only the association between cognition and psychosocial functioning, because the relations between both constructs in schizophrenia are more intricate. The interconnection of different factors (psychiatric, cognitive, and social) plays a differential role. Our data show the correlation between cognitive dysfunction and negative symptoms and revealed that negative symptoms have a more direct effect on the functional outcome than cognitive performance, the effect of which is smaller and indirect. The neurocognitive deficit and impaired social cognition could result in the reduced motivation of patients throughout the illness. These factors create a vicious cycle in schizophrenia, and we should aim to use all possible medical and behavioural interventions to minimise the impact on the daily life functioning of our patients.

Optimal everyday functioning is of utmost importance in the repertoire of interventions in patients with schizophrenia. These patients often need complex, individually tailored medical care and work support to maintain long-term social relationships and live independently. A practical assessment of the most decisive factors and their associations to improve psychosocial functioning is highly needed. Despite intensive pharmacological research, there is still a low effect size of treatment interventions to improve negative or cognitive symptoms of schizophrenia^14,66–69^. Therefore, all other evidence-based psychosocial rehabilitation approaches for the management of cognitive and negative symptoms to achieve remission and recovery are of crucial importance^70,71^.

**Limitations of the study**

1. Patients were recruited only in two centres in one country; thus the external generalisability is limited. On the other hand, the sample is big enough to demonstrate the statistical importance of the results.
2. Patients were treated with a range of different antipsychotics, and the possible extrapyramidal side effects of the medications, which could influence patients’ performance in testing, were not assessed.
3. We used the PANSS, which is a first-generation rating scale, for the assessment of negative symptoms; the use of second-generation scales could deliver more exact and reliable results. On the other hand, the PANSS has been the most widely used scale for assessing psychopathology in patients with psychosis, and at least we used the concept of PANSS negative symptoms mentioned in the EPA guidelines. The PANSS assessment unfortunately does not assess the subjects’ internal experience.
4. We did not have information about the psychosocial interventions proposed to the patients included in the study.

## Data Availability

The data used in this study can be acquired upon request. Because of restrictions based on the informed consent of the participants and GDPR, data cannot be made freely available in a public repository.

## Appendix

**Table 5:** Standardized coefficients for three models predicting PSP from the NART, length of disease, MCCB, and PANSS negative.

Term	PSP	PSP-A	PSP-B	PSP-C	PSP-D
Intercept	<0.01	<0.01	0.04	0.08	0.03
NART/CRT	0.08	−0.05	<0.02	−0.16	−0.14
Disease length (months)	−0.09	0.08	0.13	0.22^*^	0.05
MCCB (composite score)	0.09	−0.14	−0.13	0.05	−0.19
PANSS- negative	−0.64***	0.52***	0.47***	0.42***	−0.04
adj. R^2^	0.54	0.39	0.32	0.31	0.05

*MCCB* MATRICS Consensus Cognitive Battery, *NART/CRT* National Adult Reading Test/Czech Reading Test, *PANSS* Positive and Negative Syndrome Scale, PANSS negative items: Blunted Affect (N1), Poor Rapport (N3), and Lack of Spontaneity (N6); Emotional Withdrawal (N2), and Passive Social Withdrawal (N4); *PSP* Personal and Social Performance scale. ^*^*p* < .05, ^***^*p* < .001; significance adjusted by Holm correction.

Each column corresponds to a model predicting different PSP subscales (or a composite score).

## Code availability

All relevant analysis scripts are available upon request.
